# AKT mediates actinomycin D-induced p53 expression

**DOI:** 10.18632/oncotarget.1328

**Published:** 2014-01-11

**Authors:** Chih-Shou Chen, Dong-Ru Ho, Fei-Yun Chen, Chang-Rong Chen, Yu-De Ke, Jyan-Gwo Joseph Su

**Affiliations:** ^1^ Division of Urology, Department of Surgery, Chang Gung Memorial Hospital, Chiayi, Taiwan, ROC; ^2^ Department of Biochemical Science and Technology, National Chiayi University, Chiayi 600, Taiwan, ROC

**Keywords:** Actinomycin D, AKT, p53

## Abstract

At high cytotoxic concentrations, actinomycin D (ActD) blocks transcription, decreasing levels of MDM2 and thus causing p53 stabilization. At low cytostatic concentrations, ActD causes ribosomal stress, which decreases MDM2 activity, resulting in p53 stabilization and activation. ActD can thus be used for p53-based cyclotherapy. We analyzed pathways mediating ActD-induced p53 expression. Inhibitors (LY294002, wortmannin, and deguelin) of phosphatidylinositol 3-kinases (PI3K) and AKT, but not inhibitors of MEK1/2, JNK, and p38-MAPK abolished the ActD-induced p53 expression in diverse cell types. RNA interference further supported these results. When AKT was downregulated by small hairpin RNA-AKTs, ActD-induced p53 expression was significantly decreased. ActD caused AKT phosphorylation at Ser473, indicating full activation of AKT. The potential for cancer therapy is discussed.

## INTRODUCTION

In response to cellular stress such as DNA damage, oncogene activation, transcriptional inhibition, and hypoxia, tumor suppressor p53 is activated and expressed, and acts as a transcription factor to induce its target genes [1], thereby playing a central role in the regulation of DNA repair, cell cycle, apoptosis, senescence, and angiogenesis [2-4]. Its major target genes include proapoptotic genes Bax, Puma and Noxa, cell cycle regulator p21, and the senescence-inducing gene *Plasminogen activator inhibitor 1* [5]. The restriction of cellular growth by p53 has been reported to result in cell cycle arrest or apoptosis [6], and targeting p53 and restoring p53 function to limit tumor growth has been intensively researched for cancer therapy [7]. AKT is a well-known survival factor that phosphorylates and activates oncoprotein HDM2 (also known as murine double minute 2 (MDM2), HDM2 in humans), and in turn, HDM2 induces degradation of p53 [8, 9]. Thus AKT indirectly downregulates p53, and p53 negatively regulates AKT [10].

Actinomycin D (ActD), an antineoplastic antibiotic isolated from *Streptomyces* sp., has been reported to induce cytotoxicity and apoptosis, and inhibit growth of pancreatic cancer cells [11]. ActD inhibits cell proliferation by forming a stable complex with DNA duplexes via deoxyguanosine residues, resulting in the inhibition of RNA synthesis by blocking the elongation of RNA chains [12].

The application of ActD at high doses (> 800 nM) is limited due to its high toxicity through acting as a transcription blocker; however at low doses (10-100 nM) it induces p53 expression and is not highly toxic [13, 14]. In addition, at a high cytotoxic concentration (50 nM), ActD has been shown to block transcription and decrease levels of HDM2 thereby stabilizing p53 [15]. Further, at a low cytostatic concentration (2 nM), ActD causes ribosomal stress leading to a decrease in HDM2 activity and thereby p53 stabilization and activation. Therefore, combined treatment of low-dose ActD with other chemotherapeutic drugs may be a promising cancer therapy. The combined treatment of ActD with leptomycin B, a small molecule nuclear export inhibitor, has been shown to successfully lead to the accumulation of transcriptionally active p53 in the nuclei of human papillomavirus positive cervical carcinoma cells, resulting in apoptosis of the cells [16]. Due to the inhibition of RNA transcription, ActD has been found to have antineoplastic properties in the treatment of various malignant neoplasms including Wilm's tumour [14]. In addition, ActD has been shown to mimic nutlin-3 in the activation of p53-dependent transcription, induction of a reversible protective growth arrest in normal cells, and enhancement of the activity of the chemotherapeutic drugs, melphalan and etoposide, resulting in apoptosis of p53 positive human tumor cells [14].

Although low doses of ActD have been studied in p53 base cyclotherapy, the kinase pathway by which ActD induces p53 has not been examined. Cyclotherapy may be achieved by combining ActD treatment with other drugs [17, 18], and therefore understanding the cellular kinase pathway for the drugs used in combination treatment will be valuable for future cyclotherapy studies. The present study analyzed the kinase pathway through which ActD induces p53, and found that AKT was phosphorylated and activated by ActD. AKT is required in mediating ActD-induced p53 expression. Thus, there is a novel function of ActD in the upregulation of AKT-mediated p53 expression. This study clarifies the signaling pathway that induces p53 via ActD, a potential chemotherapeutic agent.

## RESULTS

### ActD dose- and time course-dependently induces protein expression and phosphorylation of p53

**Figure 1:**
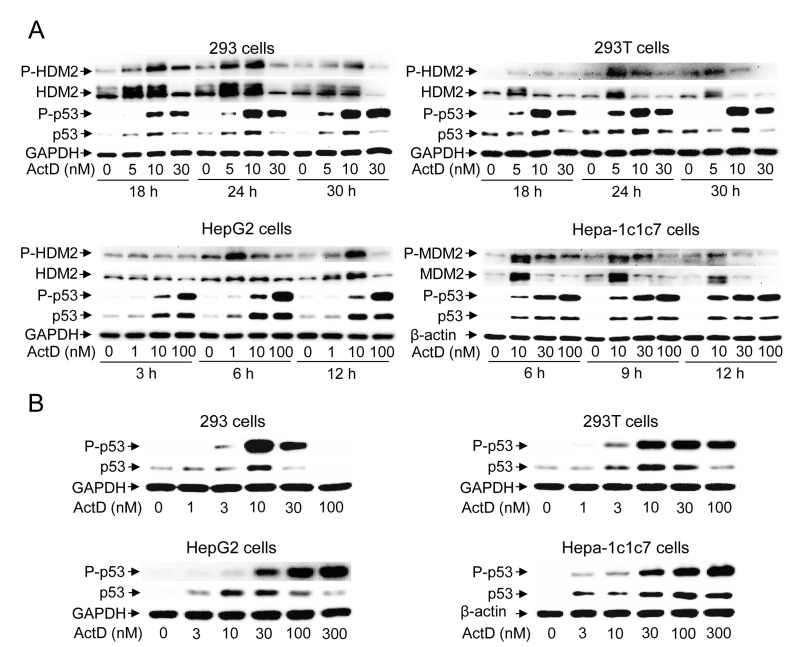
Time course and dose effect of the induction of p53 expression by actinomycin D (ActD) (A) 293 and 293T cells were treated with ActD (0, 5, 10 and 30 nM) for 18, 24, and 30 h. HepG2 cells were treated with ActD (0, 1, 10 and 100 nM) for 3, 6, and 12 h. Hepa-1c1c7 cells were treated with ActD (0, 10, 30 and 100 nM) for 6, 9, and 12 h. (B) 293 and 293T cells were treated with ActD (0, 1, 3, 10, 30 and 100 nM) for 24 h. HepG2 and Hepa-1c1c7 cells were treated with ActD (0, 3, 10, 30, 100 and 300 nM) for 6 h. The cells were then harvested, and cell lysates were analyzed by Western blotting using antibodies against p53, phospho-p53 (Ser15), HDM2, phosphor-HDM2 (Ser166), GAPDH, and β-actin.

Treatment with ActD (10 nM) distinctly induced the expression and phosphorylation of p53 at 18 h, reaching a maximal response at 24 h, and maintaining a high level of p53 for up to 30 h in the 293 and 293T cells (Fig. 1A). In contrast, treatment with ActD (10 nM) distinctly induced the expression and phosphorylation of p53 at 3 h, reaching a maximal response at 6 h, and maintaining a high level of p53 for up to 12 h in the HepG2 cells. In the Hepa-1c1c7 cells, treatment with ActD (10 nM) distinctly induced the expression and phosphorylation of p53 at 6 h, and a high level of p53 was maintained for up to 12 h (Fig. 1A). In the dosage studies, the expression and phosphorylation of p53 reached a maximal level with treatment of 10 nM ActD for 24 h in the 293 and 293T cells (Fig. 1B). In the HepG2 cells, the expression of p53 reached a maximal level with treatment of 10 nM ActD, and decreased with doses of 100 nM or higher for 6 h (Fig. 1B). Although p53 protein levels decreased after reaching the maximal level with treatment of 30 nM ActD, phosphorylation of p53 still increased with treatment of high doses (100 and 300 nM) of ActD. In the Hepa1c1c7 cells, the expression of p53 reached a maximal level with treatment of 100 nM ActD, and decreased with a dose of 300 nM for 6 h (Fig. 1B). Although p53 protein levels decreased after reaching the maximal level with treatment of 100 nM ActD, phosphorylation of p53 still increased with treatment of a high dose (300 nM) of ActD.

When the expression and phosphorylation patterns of HDM2 were examined, the results showed that HDM2 expression was induced by low doses of ActD (5 or 10 nM), and suppressed by high doses (> 10 nM) of ActD(Fig. 1A). In contrast, p53 expression was still induced at higher doses of ActD (>10 nM). The phosphorylation patterns of HDM2 were similar to the protein expression patterns of HDM2.

### ActD stimulates p53 activity

**Figure 2:**
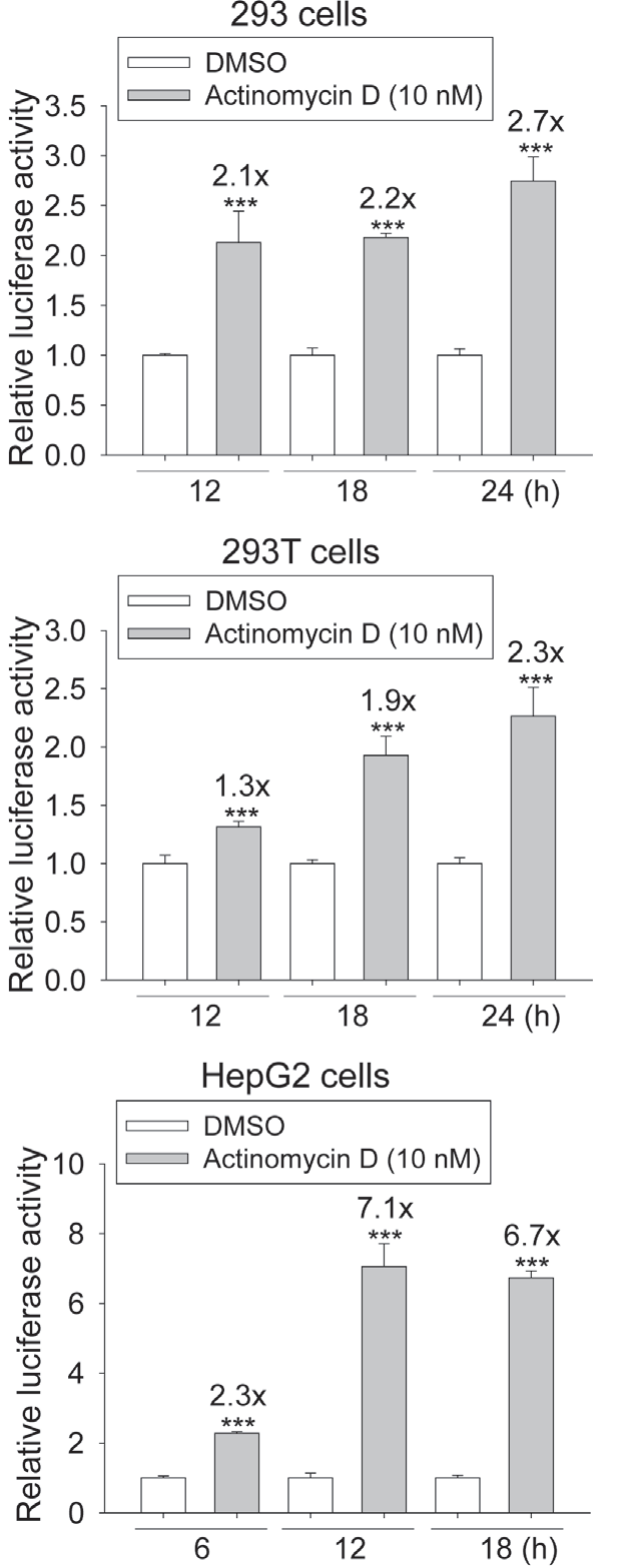
Induction of the transcriptional activity of p53 response element (p53RE) by actinomycin D (ActD) treatment 293, 293T, and HepG2 cells were transfected with plasmids of p53-TA-Luc plus RSV-*lac*Z, and then incubated with ActD (10 nM) for the indicated time periods, followed by the activity assays of luciferase and β-galactosidase. Each experiment was assayed in triplicate and repeated at least three times. *** Indicates *p* < 0.001 compared with the negative controls.

A reporter plasmid, p53-TA-Luc, was used to quantify p53 activity. The p53 activity in the 293 and 293T cells increased from 12 through 24 h after treatment with 10 nM ActD (Fig. 2). The increase in the p53 activity in the HepG2 cells was also time course-dependent and reached a plateau at 12 h after treatment with 10 nM ActD.

### The PI3K-AKT pathway mediates ActD-induced p53 expression

**Figure 3:**
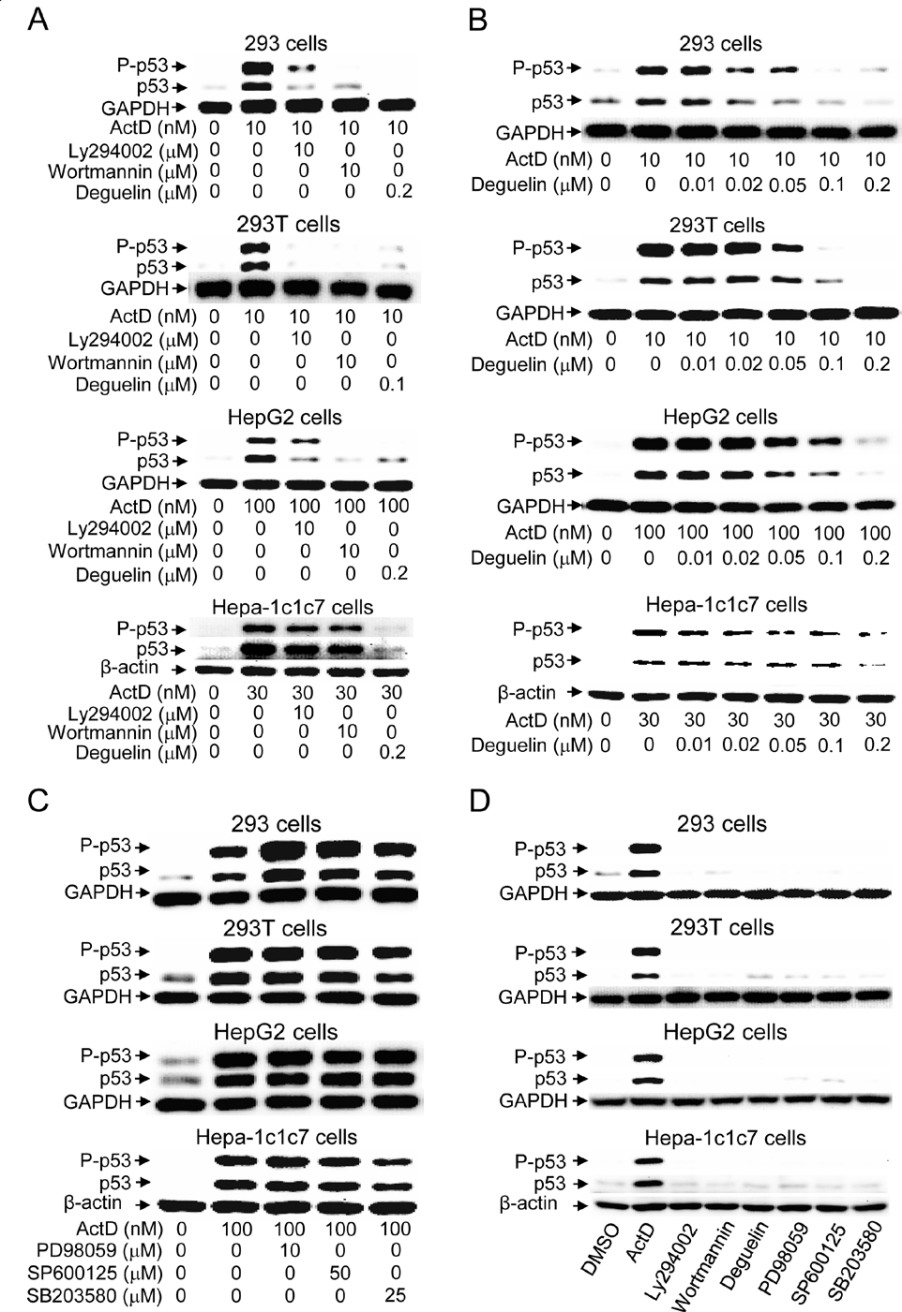
Effects of kinase inhibitors on actinomycin D (ActD)-induced p53 expression (A) Cells were pretreated with PI3K inhibitors (LY294002 (10 μM) or wortmannin (10 μM)), and an AKT inhibitor (deguelin) for 1 h, followed by treatment with ActD for 24 h in 293 and 293T cells, and for 6 h in HepG2 and Hepa-1c1c7 cells. (B) Cells were pretreated with deguelin, (0.01, 0.02, 0.05, 0.1, and 0.2 μM) for 1 h, followed by treatment with ActD for 24 h in 293 and 293T cells, and for 6 h in HepG2 and Hepa-1c1c7 cells. (C) The MEK1/2 inhibitor (PD98059, 10 μM), JNK inhibitor (SP600125, 50 μM), and p38 inhibitor (SB203580, 25 μM) were applied. Cells were pretreated with kinase inhibitors for 1 h, followed by treatment with ActD for 24 h in 293 and 293T cells, and for 6 h in HepG2 and Hepa-1c1c7 cells. (D) Cells were treated individually with ActD (10 nM), LY294002 (10 μM), wortmannin (10 μM), deguelin (0.2 μM for 293, HepG2, and Hepa-1c1c7 cells, and 0.1 μM for 293T cells), PD98059 (10 μM), SP600125 (50 μM), and SB203580 (25 μM) for 24 h in 293 and 293T cells, and for 6 h in HepG2 and Hepa-1c1c7 cells. The cells were then harvested and cell lysates were analyzed by Western blotting using antibodies against p53, phospho-p53 (Ser15), GAPDH, and β-actin.

We were interested in analyzing whether any kinase pathway mediated ActD-induced p53 expression and phosphorylation. Phosphatidylinositol-3-kinase (PI3K) inhibitors (LY294002 (10 μM) and wortmannin (10 μM)) and an AKT inhibitor (deguelin (0.1 or 0.2 μM)) were added to the 293, 293T, HepG2, and Hepa-1c1c7 cells that had been treated with ActD. All of these inhibitors either abolished or highly decreased the ActD-induced p53 expression (Fig. 3A). Deguelin dose-dependently suppressed p53 expression, and 0.05 μM deguelin decreased the p53 expression in the 293, 293T and HepG2 cells (Fig. 3B). However, 0.2 μM deguelin was required to cause a distinct decrease in p53 expression in the Hepa-1c1c7 cells. Inhibitors of mitogen-activated protein kinases (MAPKs), including MEK1/2 inhibitor (PD98059) and Jun N-terminal kinase inhibitor (SP600125), did not suppress the ActD-induced p53 expression or phosphorylation (ser15) (Fig. 3C). p38 inhibitor (SB203580) caused a minor reduction in the ActD-induced increase in the expression and phosphorylation of p53 in the 293T and Hepa-1c1c7 cells, but not in the 293 and HepG2 cells. The different results for the p38 inhibitor may be due to cell-specific and species-specific factors. None of the applied kinase inhibitors themselves distinctly induced p53 expression (Fig. 3D).

**Figure 4:**
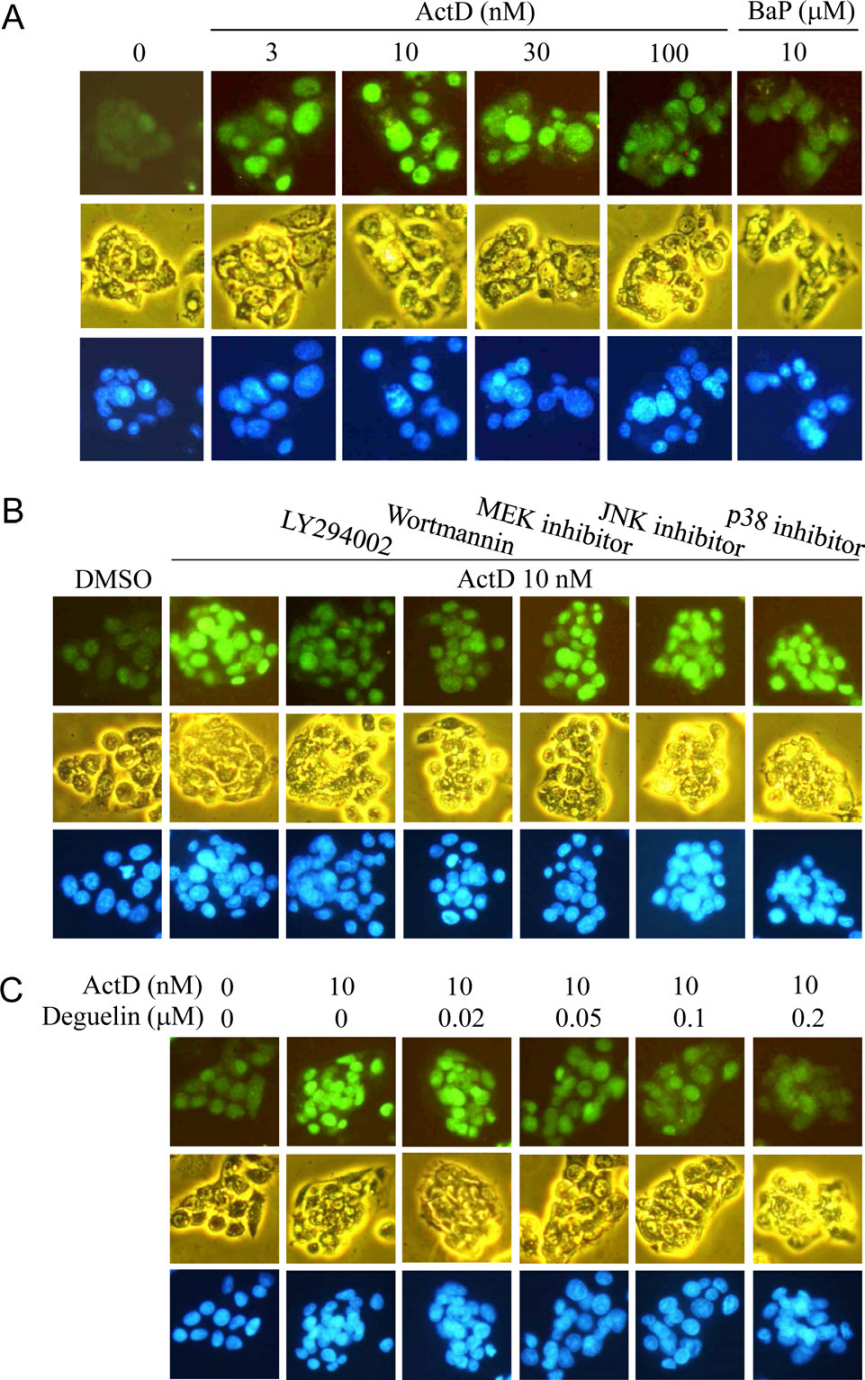
Actinomycin D (ActD)-induced p53 expression as revealed by immunofluorescence imaging HepG2 cells were seeded on microscope cover glasses in 6-well plates overnight before drug treatment. (A) Cells were treated with ActD (3, 10, 30, and 100 nM) and benzo[*a*]pyrene (BaP) (10 μM) for 6 h. (B) The PI3K inhibitors, (LY294002 (10 μM) and wortmannin (10 μM)), MEK1/2 inhibitor (PD98059, 10 μM), JNK inhibitor (SP600125, 50 μM), and p38 MAPK inhibitor (SB203580, 25 μM) were applied. Cells were pretreated with kinase inhibitors for 1 h, followed by treatment with ActD for 6 h in HepG2 cells. (C) Cells were pretreated with AKT inhibitor (deguelin, 0.02, 0.05, 0.1, and 0.2 μM) for 1 h, followed by treatment with ActD (10 nM) for 6 h. The cells were then fixed with ethanol. The expression of the p53 protein was probed using an antibody against p53, as revealed by fluorescence of goat polyclonal secondary antibody to mouse IgG-H&L (DyLight® 488). The fluorescence emitted by the cells was viewed using a fluorescence microscope, equipped with U-MWB2 optical filters at excitation/emission wavelengths of 460~490/520 nm. Nuclei were stained with Hoechst 33342 (5 μg/ml) and observed by a fluorescence microscope equipped with U-MWU optical filters with a U-MWU optical filter at an excitation wavelength of 355 nm and an emission wavelength of 420 nm. The morphologies of the cells were examined by phase-contrast microscopy.

**Figure 5:**
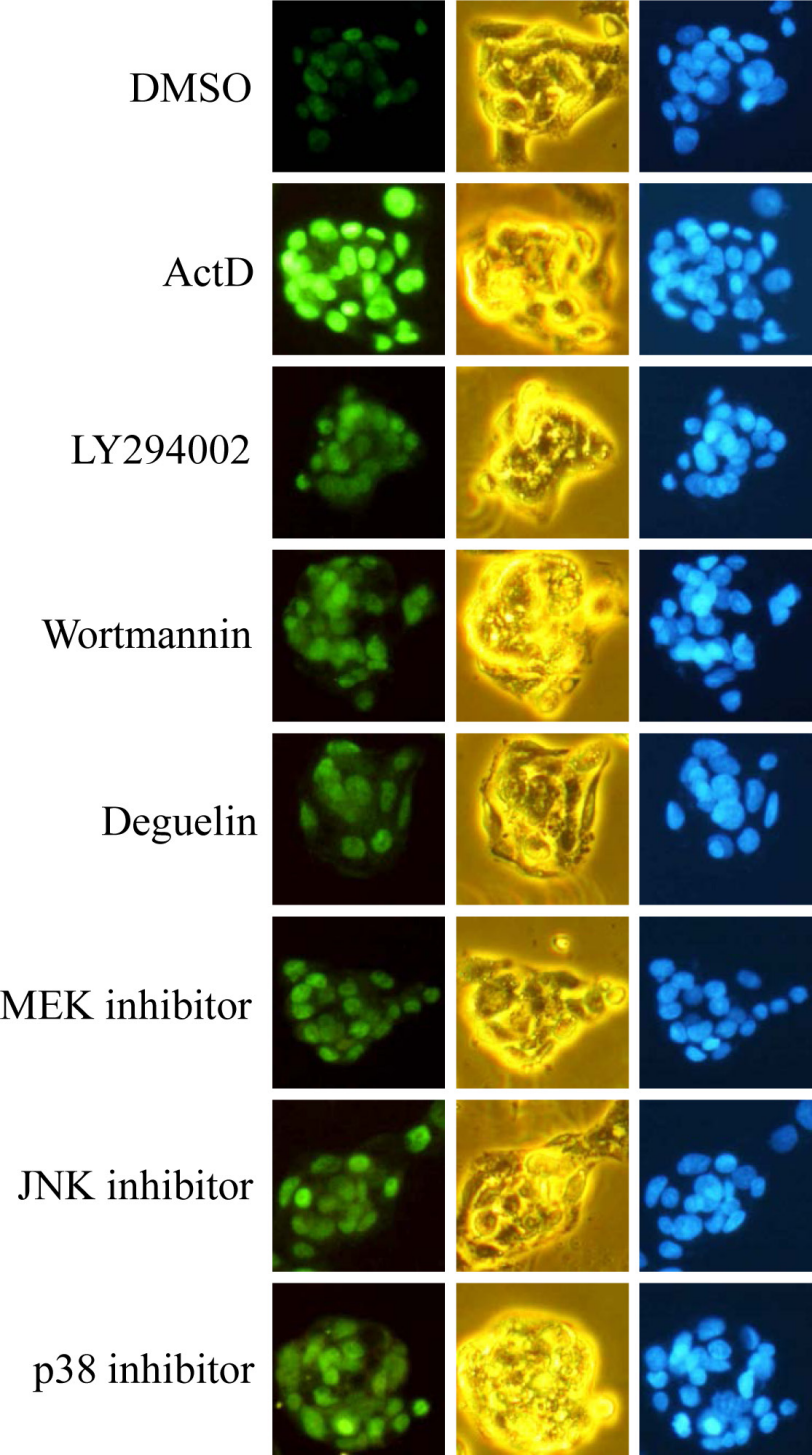
Effects of kinase inhibitors on p53 expression as revealed by immunofluorescence imaging HepG2 cells were seeded on microscope cover glasses in 6-well plates overnight before drug treatment. Cells were treated individually with ActD (10 nM), LY294002 (10 μM), wortmannin (10 μM), deguelin (0.2 μM), PD98059 (10 μM), SP600125 (50 μM), and SB203580 (25 μM) for 6 h in HepG2 cells.

In order to further confirm the results of Western blotting, the expression of p53 was further examined by immunofluorescence staining. The HepG2 cells were treated with ActD for 6 h, and the p53 expression level was examined by immunofluorescence imaging. The benzo[*a*]pyrene (BaP)-induced p53 expression was used as a positive control, and the blue fluorescence dye, Hoechst 33342, revealed the location of the nuclei. Fluorescence was dose-dependently increased by treatment with ActD (3-30 nM), but decreased by treatment with 100 nM ActD (Fig. 4A). These results were similar to those derived from Western blotting (Fig. 1B). PI3K inhibitors (LY294002 (10 μM) and wortmannin (10 μM)), but not MEK1/2 inhibitor (PD98059), JNK inhibitor (SP600125), or p38 MAPK inhibitor (SB203580), inhibited the ActD-induced p53 expression in the immunofluorescence assays (Fig. 4B). A dose of 50 nM and higher of deguelin suppressed the ActD-induced p53 expression in the immunofluorescence assays (Fig. 4C). These results were also similar to those derived from Western blotting (Fig. 3B and C). When the cells were treated with each kinase inhibitor individually, the applied kinase inhibitors themselves caused a minor induction of p53 expression (Fig. 5).

### AKT is needed in the ActD induction of p53 expression

To determine whether AKT was needed in the ActD induction of p53 expression, shRNAs for AKT1 were introduced into the HepG2 and Hepa-1c1c7 cells by lentiviruses to knock down AKT levels. Lentiviruses were used to individually produce the shRNAs of the green fluorescence protein (GFP), firefly luciferase (LUC), three different shRNAs of human AKT1 (AKT1-a, -b, and -c), and two different shRNAs of mouse Akt1 (Akt1-1 and−2) in the virus-infected cells. Viruses with shRNA-LUC and shRNA-GFP were used as the controls of viral infection.

**Figure 6:**
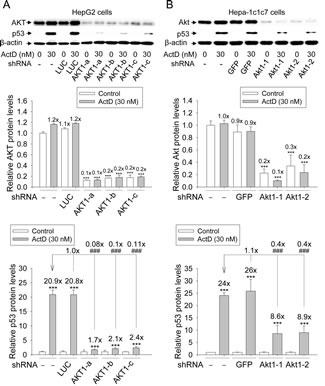
Effects of AKT RNAi on actinomycin D (ActD)-induced p53 expression (A) HepG2 cells, with and without shRNA-LUC and shRNA-AKT1 were treated with ActD (30 nM) for 6 h. (B) Hepa-1c1c7 cells with and without shRNA-GFP and shRNA-Akt1 were treated with ActD (30 nM) for 6 h. The AKT and p53 protein levels revealed by Western blotting were quantified and standardized against the amount of β-actin protein. The results are expressed as the mean ± SD (n=3). ***p<0.001 and ^###^*p*<0.001. * A comparison with DMSO-treated cells without shRNA. ^#^ A comparison with ActD-treated cells without shRNA.

The relative AKT and p53 protein levels in the cells with and without shRNA-LUC, shRNA-GFP, and shRNA-AKT1, were demonstrated by Western blotting. There was no significant difference in AKT protein level between the cells without shRNA and those with shRNA-LUC or shRNA-GFP (Fig. 6A and B). Only 10-20% and 10-30% of AKT was left in HepG2 and Hepa-1c1c7 cells with shRNA-AKT, respectively.

ActD (30 nM) highly induced p53 protein in the cells without shRNA and in the cells with shRNA-LUC and shRNA-GFP (Fig. 6A and B). In contrast, the ActD-induced p53 protein levels in the HepG2 and Hepa-1c1c7 cells with shRNA-AKT were only 8-11% and 40%, respectively, of that in the cells without shRNA (Fig. 6A and B).

### ActD induces phosphorylation of AKT

**Figure 7:**
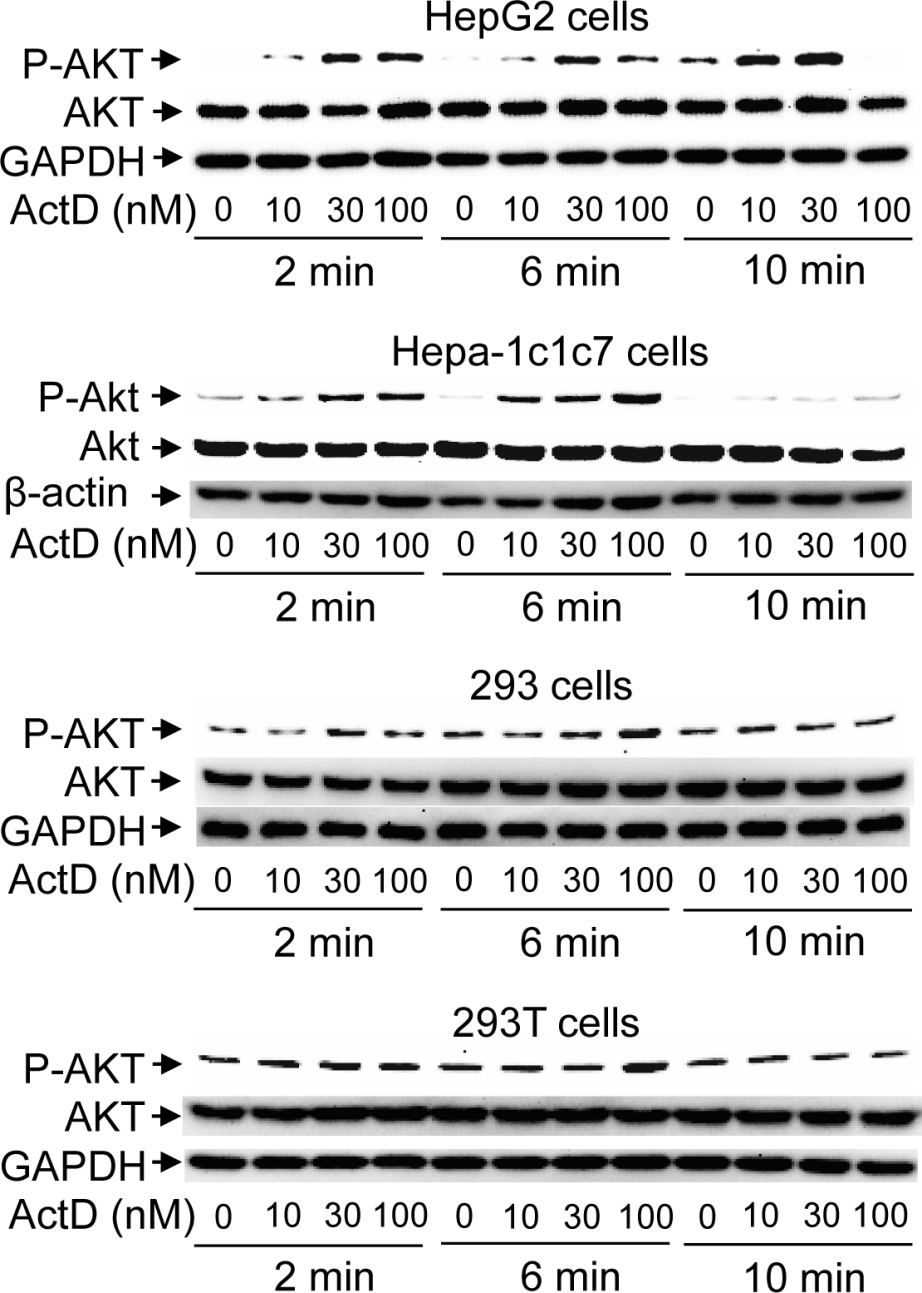
Phosphorylation of AKT induced by actinomycin D (ActD) (A) 293, 293T, HepG2, and Hepa-1c1c7 cells were treated with ActD (0, 10, 30 and 100 nM) for 2, 6, and 10 minutes (min). The cells were then harvested, and cell lysates were analyzed by Western blotting using antibodies against Akt and anti-phospho-Akt (Ser473), GAPDH, and β-actin.

In order to further identify whether the ActD-induced p53 expression was mediated by the AKT pathway, phosphorylation at serine 473 of AKT was analyzed. Treatment with 10 nM ActD for 2 and 6 minutes distinctly phosphorylated AKT at serine 473 in the HepG2 and Hepa-1c1c7 cells, however 30 and 100 nM ActD was required for distinct phosphorylation of AKT in the 293 and 293T cells, respectively (Fig. 7). Phosphorylation of AKT decreased after treatment with ActD for 10 minutes in the 293, 293T, and Hepa-1c1c7 cells, but still increased after treatment with ActD (10 and 30 nM) for 10 minutes in the HepG2 cells.

### ActD decreases cell viability

**Figure 8:**
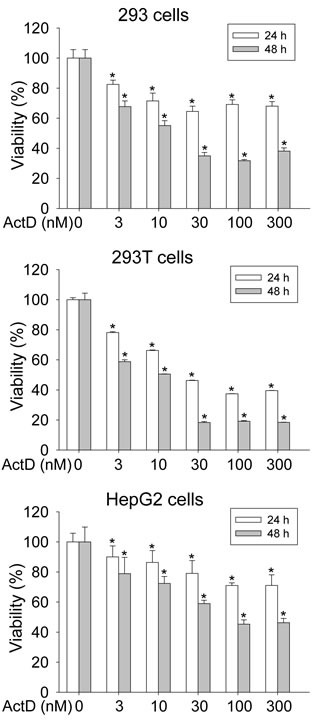
Influence on cell viability by actinomycin D (ActD) 293, 293T, and HepG2 cells were treated with ActD for 24 and 48 h. Cell viability was analyzed by a MTT assay. Data are expressed as the mean ± SD, *n* = 6. * *p* < 0.05.

A cell viability assay was then performed. Treatment with ActD at low doses of 3-30 nM for the 293 and 293T cells, and 3-100 nM for the HepG2 cells, for 24 and 48 hours dose-dependently decreased cell viability (Fig. 8). However, treatment with ActD of 30-300 nM for the 293 and 293T cells, and 100 and 300 nM for the HepG2 cells, for 24 and 48 hours significantly decreased cell viability by a similar level.

## DISCUSSION

A high dose of ActD has been reported to cause DNA damage and suppress transcription from all three classes of RNA polymerases, and ActD at a low dose has been reported to selectively block RNA poly I-dependent transcription [19]. However, ActD has been shown to induce p53 expression through ribosomal stress [15, 20]. It has also been reported that the ribosomal protein L11 interacts with oncoprotein HDM2 and inhibits the function of HDM2, thus leading to the stabilization and activation of p53 [20-22]. In addition, the interaction of L11 with HDM2 has been shown to be enhanced in response to ActD [19, 21]. ActD at a low dose has been reported to induce p53, and in turn, p53 increase the expression of HDM2 [15]. HDM2 has been shown to be transcribed by p53, which forms a negative feedback loop to promote poly-ubiquitination and degradation of p53 [23]. Although HDM2 is induced indirectly by ActD, ActD also induces the interaction of L11 with HDM2 leading to inhibition of HDM2 function. Therefore p53 induced by ActD is more stable than that induced by other drugs without inhibiting HDM2 function. Accordingly, it is worth exploring the role of ActD as a p53 activator to reduce the growth of tumors.

Although it has been reported that ActD induces p53 expression, the kinase pathway for ActD-induced p53 has not been identified. Our results showed that inhibitors of PI3K and AKT, but not inhibitors of MEK1/2, JNK or p38, abolished ActD-induced p53 expression. MEK1/2 is immediately upstream of extracellular signal-regulated kinase (ERK) 1/2, and has been reported to activate ERK1/2 [24]. When a MEK1/2 inhibitor was applied, ERK1/2 activity was also inhibited. These kinase inhibitors of MEK1/2, JNK or p38 themselves did not distinctly induce p53 expression. The Western blotting results were further confirmed by *in situ* immunofluorescence staining. Phosphorylation of Ser15 in p53 is a sign that p53 is activated [25]. It has been shown that the MAP kinases including JNKs, ERKs, and p38 phosphorylate p53 in response to different stressful stimuli, and in turn, activate a p53 response leading to cell cycle arrest and apoptosis [24]. In addition, Ser15 of p53 has been shown to be phosphorylated by JNKs, ERKs, and p38 [24]. However, the ActD-induced phosphorylation (Ser15) of p53 was not inhibited by the inhibitors of p38, JNKs and ERKs in the current study, indicating that the phosphorylation (Ser15) of p53 was independent of these three MAP kinases.

Serine/threonine kinase AKT is a downstream kinase of PI3K and a critical PI3K effector [26]. AKT is recruited to plasma membranes by PtdIns(3,4,5)P3 (PIP3), and AKT is then phosphorylated on Thr-308 and Ser473 by PDK1 and mTORC2-Rictor, respectively. Upon Ser473 phosphorylation by mTORC2, AKT is fully activated [27]. Our results showed that ActD induced the phosphorylation of AKT at Ser473 within 2 minutes. In addition to PI3K inhibitors, deguelin, an AKT inhibitor, suppressed ActD-induced p53 expression in a dose-dependent manner. This confirms that ActD-induced p53 expression is mediated by the activation of AKT. Finally, the necessity of AKT in mediating ActD-induced p53 expression was confirmed by RNA interference on AKT. ActD-induced p53-expression significantly decreased when AKT expression was knocked-down by shRNA. Although AKT has been shown to downregulate p53 [28], the findings in the current study reveal a new function of AKT in the upregulation of p53.

PI3K-AKT signaling has been reported to promote the activation of the oncoprotein HDM2 and to downregulate the p53 tumor suppressor [28], and therefore AKT is defined as a survival factor. In contrast, AKT has been shown to be downregulated by p53 [10, 29]. However, our results showed that treatment with ActD immediately activated AKT signaling, and thereby induced p53 expression.

MAPKs have been widely reported to be involved in the activation of p53. The p38-mediated p53 phosphorylation at Ser15 and subsequent p53 induction has been reported to be responsible for apoptosis induced by nitric oxide, hypoxic conditions, DNA-damaging agents, 1-nitropyrene, and benzo[*a*]pyrene [30-32]. In response to oxidative stress, JNKs have been shown to phosphorylate p53 at Ser15 [33], and ERKs have been shown to mediate the p53 activation induced by colcemid and resveratrol [34, 35]. AKT is a central point in cell signaling downstream of growth factors, cytokines, and other cellular stimuli, and thus it acts as a key regulator of a wide range of cellular processes including growth, proliferation and survival [9]. Therefore, AKT is best known for promoting cell survival and growth. In contrast to MAPKs, AKT inhibits the expression and function of apoptotic proteins, BAD and caspase-9, and promotes the activity of HDM2 which degrades p53 [9]. It has been reported that AKT and p53 counteract each other [36-42]. In contrast, the results of the current study showed that AKT, but not MAPKs, mediated ActD-induced p53 expression and activation.

With regards to the induction of p53, the p53 protein expression peaked at 24 h, 24 h, 6 h, and 6 h with 10 nM, 10 nM, 10, and 100 nM ActD treatment in the 293, 293T, HepG2, and Hepa-1c1c7 cells, respectively. Upon longer periods and higher doses of ActD the p53 expression decreased, which may be due to suppression of transcription from all three classes of RNA polymerases. Activated p53, a sequence-specific transcription factor, has been reported to bind to the p53RE on p53-targeted genes such as p21, to direct its downstream signals and actions [43]. In the current study, ActD also showed a time course-dependent increase in p53 activity, assayed by measuring the transcriptional activity of a p53RE reporter plasmid. Interestingly, ActD-induction of p53 expression was much faster in liver cells than in kidney cells.

HDM2 was transcribed by the p53 transcription factor, and the patterns of expression and phosphorylation of HDM2 were parallel to those of p53 at low doses (5 or 10 nM) of ActD treatment. However, p53, but not HDM2 expression was still induced by high doses (> 10 nM) of ActD treatment. Further studies are needed to elucidate the discrepancy between these expression patterns at high doses of ActD treatment.

ActD (≤ 10 nM) dose-dependently decreased cell viability, and its induction of active p53 also peaked at 10 nM in the 293 and 293T cells. High doses (≥ 30 nM) of ActD did not further decrease cell viability or increase p53 expression. In contrast, ActD (≤ 100 nM) dose-dependently decreased cell viability and increased active p53 in the HepG2 cells. Therefore, the results of the cell viability assay reflected the activation of p53.

It has been reported that the activation of AKT involves ionizing radiation induction of p53 [44]. Constitutively expressed active myristoylated AKT has been reported to increase p53 levels [45]. However, ActD is the first chemical compound that has been shown to date to induce the AKT-mediated p53 expression.

An ideal chemotherapeutic agent is expected to specifically kill tumor cells, cause the least amount of undesirable side effect, and leave normal cells unharmed. However, the specificity of conventional chemotherapeutic drugs towards cancer cells is limited. Cyclotherapy is a prospective therapeutic strategy for the protection of normal cells from the side effects of chemotherapy [17]. The idea of p53-based cyclotherapy is that a p53 activator ceases proliferation in normal tissues while leaving the p53-deficient tumor susceptible to the toxicity of S- or M-phase chemotherapeutic poisons [18]. In addition, the p53 activator does not affect the sensitivity of tumor cells to the chemotherapeutic agents.

ActD has been shown to have a reversible cytostatic effect and the ability to arrest cells in both the G1 and G2 phase of the cell cycle [17]. A low, non-genotoxic, dose (about 1 nM) of ActD has been shown to induce a reversible cytostatic effect in normal proliferating dermal fibroblasts and protect them from the aneuploidy induced by the aurora kinase inhibitor VX-680 and the toxic effects of gemcitabine [46, 47]. In contrast, pretreatment of ActD did not weaken the growth inhibitory effects of VX-680 in clonogenic assays [46] and did not affect the sensitivity of the tumor cells to gemcitabine [47].

ActD is a classic clinically approved drug [46]. Based on studies on ActD, about 1-4 nM of ActD is suggested for cyclotherapy [18, 46, 47], and 3 nM or higher may be appropriate for chemotherapy.

In summary, our findings showed that ActD induces the phosphorylation of AKT, thereby activating AKT. In turn, AKT signaling is essential in mediating ActD-induced p53 expression. The expression and phosphorylation (Ser15) of p53 is independent of JNKs, ERKs, and p38. Thus, upon activation by ActD, AKT becomes an inducer of p53 tumor suppression instead of being a survival factor, as consistently shown in human embryonic kidney cell lines (293 and 293T), human hepatoma cell line (HepG2), and mouse hepatoma cell line (Hepa-1c1c7). These findings on ActD signaling to induce p53 may be valuable for the development of treatment for human tumors.

## MATERIALS AND METHODS

### Reagents and antibodies

ActD, LY-294002, wortmannin, deguelin, PD98059, and Hoechst 33342 were obtained from Sigma (St. Louis, MO), and SB203580 and SP600125 were obtained from Selleckchem (Houston, TX). Minimum essential medium alpha (MEMα), MEM, Dulbecco's modified Eagle medium/nutrient mixture F12 (DMEM/F12), and fetal bovine serum (FBS) were obtained from Gibco (Grand Island, NY). Goat anti-mouse immunoglobulin G (IgG)-horseradish peroxidase (HRP), and goat anti-rabbit IgG-HRP were obtained from Santa Cruz Biotech (Santa Cruz, CA). Anti-glyceraldehyde 3 phosphate dehydrogenase (GAPDH), anti-p53, and goat polyclonal secondary antibodies to mouse IgG-H&L (DyLight® 488) were obtained from Abcam (Cambridge, UK). Anti-phospho-p53 (ser15), antiphospho-HDM2 (ser166), anti-Akt and anti-phospho-Akt (Ser473) were obtained from Cell Signaling (Danvers, MA), and anti-HDM2 was obtained from Calbiochem (San Diego, CA). Serine/threonine phosphatase inhibitor cocktail was obtained from Bionovas (Toronto, Ontario). T-Pro Non-liposomal Transfection Reagent II (NTRII) was obtained from JF Ji-Feng Biotechnology (Taipei, Taiwan). Fluoromount-G was obtained from Southern Biotech (Birmingham, Alabama).

### Cell cultures

293 is a human embryonic kidney cell line, and 293T is a derivative of 293 cells with an expression of SV40 T antigen. HepG2 is a human hepatoma cell line, and Hepa-1c1c7 is a mouse hepatoma cell line. The 293 and Hepa-1c1c7 cells were maintained in MEMα, and the HepG2 cells in MEM plus 10% heat-inactivated FBS, 2 mM L-glutamine, 100 units/ml penicillin, and 100 μg/ml streptomycin. The 293T cells were maintained in DMEM/F12 plus 5% heat-inactivated FBS, 2 mM L-glutamine, 100 units/ml penicillin, and 100 μg/ml streptomycin. The cultured cells were kept at 37°C in a 95% air/5% CO_2_ environment. The agents were dissolved in dimethyl sulfoxide (DMSO).

### Plasmid construction and reporter activity assay

p53-TA-Luc contains a p53 response element (p53RE), located upstream of the minimal TA promoter (Clontech, Mountain View, CA). The other reporter (RSV-*lac*Z) expresses a *lac*Z gene-encoded β-galactosidase, driven by a Rouse sarcoma virus long terminal repeat (LTR). Luciferase activity indicates transcriptional activity of the p53RE, and β-galactosidase activity of RSV-*lac*Z was used to normalize the luciferase activity. For DNA transfection, the HepG2, 293, and 293T cells were seeded respectively at 2 × 10^5^, 2 × 10^5^, and 1 × 10^5^ cells/well in 24-well plates overnight, and then the p53-TA-Luc luciferase reporter plasmid and RSV-*lac*Z plasmids were transiently transfected into the cells using NTRII for 6 h, followed by treatment with the test compounds. Cell lysates were collected at the appropriate time points after treatment with the test compounds and were assayed for both luciferase and β-galactosidase activities using Britelite (PerkinElmer) and a Galacto-Star™ System (Tropix, Bedford, MA), respectively, according to the manufacturer's instructions.

### Western blotting

The HepG2, Hepa-1c1c7, 293, and 293T cells were seeded respectively at 1 × 10^6^, 4 × 10^5^, 1.5 × 10^6^, and 1.3 × 10^6^ cells/6-cm dish overnight. The cells were then cultured with the test compounds for the appropriate time periods. At the end of the desired treatment times, cell lysates were prepared in lysis buffer (1% NP-40, 0.5 mM Tris-HCl (pH 7.5), 0.14 M NaCl, 5 mM KCl, 5 mM EDTA, and 1 mM phenylmethylsulfonyl fluoride) plus serine/threonine phosphatase inhibitor cocktail. Western blotting was performed as described previously [48].

### Immunofluorescence staining

To detect the expression of p53, the HepG2 cells were seeded at 1 × 10^6^ cells/well on microscope cover glasses in 6-well plates overnight before being treated with the drugs for the appropriate time periods, followed by washing with phosphate-buffered saline and fixing with ethanol. The expression of the p53 protein was probed using an antibody against p53, as revealed by fluorescence of a goat polyclonal secondary antibody to mouse IgG-H&L (DyLight® 488). The bisbenzimide dye, Hoechst 33342 (5 μg/ml), was then added to stain the chromosomes and reveal the location of the nuclei, and Fluoromount-G was added to reduce fluorochrome quenching during analysis of the slides by fluorescence microscopy. Fluorescence emitted by DyLight® 488 was viewed using a fluorescence microscope (Olympus, Tokyo, Japan), equipped with U-MWB2 optical filters at excitation/emission wavelengths of 460-490/520 nm. The fluorescence emitted by Hoechst 33342 was viewed using a fluorescence microscope, equipped with U-MWU optical filters at excitation/emission wavelengths of 330-385/420 nm.

### RNA interference (RNAi)

To perform RNAi and knock down AKT, 21 nucleotide duplexes corresponding to the human AKT1 mRNA (GenBank: NM_005163) and the mouse Akt1 mRNA (GenBank: NM_009652) were carried individually by lentiviruses (National RNAi Core Facility, Taipei, Taiwan). The three nucleotide duplexes for human AKT1 were AKT1-a (GCATCGCTTCTTTGCCGGTAT, clone ID: TRCN0000221554), AKT1-b (GATCCTCAAGAAGGAAGTCAT, clone ID: TRCN0000221553), and AKT1-c (CGCGTGACCATGAACGAGTTT, clone ID: TRCN0000221555). The two nucleotide duplexes for mouse Akt1 were Akt1-1 (TCTGAGACTGACACCAGGTAT, clone ID: TRCN0000022937) and Akt1-2 (GCACATCAAGATAACGGACTT, clone ID: TRCN0000022934).

Nucleotide duplexes for luciferase (LUC; CAAATCACAGAATCGTCGTAT, clone ID TRCN0000072246) and green fluorescence protein (GFP; TATCATGGCCGACAAGCA, clone ID: TRCN0000072180) were used as controls for viral infection. HepG2 cells (1.6 × 10^5^ cells/well) and Hepa-1c1c7 (1 × 10^5^ cells/well) cells were seeded individually in 6-well plates overnight and then infected by a lentivirus (4 × 10^5^/well) for 24 h. Forty-eight hours after infection, the HepG2 and Hepa-1c1c7 cells with shRNA were selected by 2 and 1 μg/ml puromycin, respectively, for 12 h to obtain stable infectants. The cells were then maintained in a medium containing puromycin (0.5 μg/ml).

### Determination of cell viability by MTT assay

HepG2, 293, and 293T cells were seeded in 96-well plates at 4 x 10^3^, 1 x 10^4^, and 1 x 10^4^ cells, respectively, with 0.1 ml medium. After overnight culture, they were treated with a range of concentrations of ActD for 24 and 48 h, followed by incubation with methylthiazolyldiphenyl-tetrazolium bromide (MTT) (Sigma) for the assay. The optical density was detected at 550 nm using an enzyme-linked immunosorbent assay plate reader (BIO-TEK, Winooski, VT). Six samples were assayed for each experiment which was repeated at least three times.

### Statistical analysis

Data are representative of at least three independent experiments under identical conditions and are expressed as the mean ± standard deviation (SD). Differences in the data of the controls and further treatment with various compounds were analyzed using the Student's *t*-test. Statistical probability (*p*) was expressed as * *p* < 0.05, *** *p* < 0.001 and ^###^ *p* < 0.001. Means were considered significantly different at *p* < 0.05.
